# The impact of parastomal hernia on quality of life using data from the CIPHER prospective cohort study

**DOI:** 10.1007/s10198-025-01768-5

**Published:** 2025-03-11

**Authors:** Joel Glynn, William Hollingworth, Jessica Harris, Syed Mohiuddin, Lucy Ellis, Barnaby C. Reeves, Neil Smart, on behalf of the CIPHER study group

**Affiliations:** 1 Health Economics and Health Policy (HEHP), Bristol Medical School, University of Bristol, Bristol, UK; 2 Bristol Trials Centre, Bristol Medical School, University of Bristol, Bristol, UK; 3 Science, Evidence and Analytics, National Institute for Health and Care Excellence (NICE), London, UK; 4 Royal Devon University Healthcare NHS Foundation Trust, Exeter, UK

**Keywords:** I10, Parastomal hernia, Stoma, Health-related quality of life, EQ-5D-5L, Background

## Abstract

**Objectives:**

Despite being a common side effect of stoma surgery, little is known about the health-related quality-of-life (HRQoL) impact of parastomal hernia (PSH). We studied the association between HRQoL and self-reported PSH using data from the large CIPHER prospective cohort study of patients living with a stoma.

**Methods:**

Over 12 months, HRQoL was captured at up to four time points for 2,341 individuals with stomas using EuroQol-5D-5L (EQ-5D-5L). Applying a repeated measures regression, we analysed the association between HRQoL and the incidence of self-reported PSH in the year following surgery. Using ordinal regressions, we estimated the odds of reporting worse function in each of the five EQ-5D-5L dimensions among those reporting PSH. We estimated the average number of quality-adjusted life days (QALDs) lost in those reporting PSH.

**Results:**

Patients experiencing PSH reported significantly lower EQ-5D-5L scores at 12 months following stoma formation (−0.099 [95%CI: −0.126 to −0.071]), amounting to 22.3 QALDs lost per-person-per year. Patients reporting PSH at 12 months had more problems in all EQ-5D-5L dimensions. In four of five dimensions, patients with PSH had more than double the odds of reporting worse HRQoL levels; the difference was most substantial for pain/discomfort (odds ratio 2.80 [95%CI: 2.17 to 3.62]).

**Conclusion:**

Developing PSH significantly reduces HRQoL across a range of health outcomes, including pain/ discomfort, usual activities, self-care, mobility, and anxiety/depression. Therefore, developing and evaluating surgical techniques to prevent PSH is important to reduce the prevalence of PSH following stoma formation. Estimates of HRQoL presented here can be used in cost-effectiveness studies evaluating such interventions.

**Supplementary Information:**

The online version contains supplementary material available at 10.1007/s10198-025-01768-5.

A stoma is a surgically created opening in the abdomen, where the contents of the bowel are diverted outside the body usually into a pouch [1, 2]. Colostomy or Ileostomy surgery may be indicated in several conditions that affect the bowel including benign and malignant tumours, inflammatory bowel diseases (IBD), and diverticular disease. Stomas can be temporary, allowing time for the bowel to heal following surgery, or permanent. Colostomy surgery has been estimated to cost between £6,000 and £7,000 per patient [3] and requires associated stoma appliances and accessories amounting to between £780 and £2,300 per patient per year [4]. Across high-income countries, it is estimated that 1 in 500 people are living with a stoma. In the UK, this amounts to 100,000 people with approximately 20,000 additional stomas created every year [5].

A frequent side effect of living with a stoma is the development of a parastomal hernia (PSH). PSH is defined by the European Hernia Society (EHS) as an “abnormal protrusion of the contents of the abdominal cavity through the abdominal wall defect created during placement of a colostomy, ileostomy or ileal conduit stoma” [6]. The incidence of PSH amongst individuals with a stoma is not precisely known. Estimates from relatively small cohort studies vary greatly from as low as 30% all the way to 94% [7, 8]. This variation may be due to the lack of a standardised PSH diagnostic method; ultrasound, computed tomography (CT), patient report, and clinical examination have all been used in the literature [9]. Studies had variable follow-up times and although PSH is common in the first few years after stoma formation, it has been reported to occur up to 20 years postoperatively [10]. Stoma populations are heterogeneous, varying by indication for surgery, type of stoma formed (e.g., ileostomy, colostomy), permanence, and other aspects of the stoma types (e.g., end, loop).

Approximately three-quarters of individuals who develop PSH experience clinical symptoms [11]. PSH is associated with reduced physical functioning, greater pain, and increased embarrassment and shame [12]. The likelihood of PSH may be reduced at the index stoma surgery using mesh or other prophylactic measures [13]. PSH can also be treated once it has developed through surgical repair [14, 15]. It is important to test the cost-effectiveness of these interventions. However, robust estimates of the impact of PSH on health-related quality of life (HRQoL) and quality-adjusted life years (QALYs) are not currently available.

The UK Cohort Study to Investigate the Prevention of Parastomal Hernia (CIPHER) aims to establish the incidence of symptomatic and radiologically confirmed PSH up to 4 years after stoma surgery [16]. It also evaluates the effects of key technical surgical steps during stoma formation on the risk of subsequent PSH [16]. The CIPHER study collected HRQoL data in a large and representative sample of the stoma population. In this paper, we estimate the impact of PSH on QALYs and specific domains of HRQoL.

## Method

### CIPHER study

Data used in this analysis were collected in the CIPHER multicentre prospective cohort study [16]. The project was granted ethical approval on the 8th of November 2017 (REC reference: 17/WM/0401). Full details of the CIPHER study protocol have been published elsewhere [16].

Participants were eligible for CIPHER if they were aged 18 or older and were undergoing any intervention classified as expedited or elective surgery (using the definition of the National Confidential Enquiry into Patient Outcome and Death), with the intention of forming a permanent or temporary stoma [17]. Participants were excluded if they had undergone emergency surgery, had a previous abdominal wall stoma, had surgery with the aim of forming a double-barrelled stoma or urostomy, or had a life expectancy of less than 12 months. Recruitment began in December 2017 and ended on June 2021. Eligible patients were given an information leaflet and asked to provide written consent before participating. In approximately 30% of cases, written consent was obtained retrospectively, up to 6 weeks post-surgery.

### Sample and data collection

Our analysis included all participants recruited to CIPHER who had completed at least one HRQoL questionnaire at baseline or follow-up and were alive 12 months post-surgery. Demographic and clinical data were collected on case report forms (CRFs) at recruiting hospitals. Self-reported HRQoL was collected using EQ-5D-5L questionnaires [18]. EQ-5D-5L is a health-related quality-of-life measure that is designed for use across a wide range of health conditions. The questionnaire comprises five dimensions; mobility, self-care, usual activities, pain/discomfort, and anxiety/depression. Each of these dimensions has 5 response levels ranging from no problems (level 1) to extreme problems (level 5). Responses are converted to an utility score using values derived from surveys of the general population. These values reflect population preferences for every response permutation or ‘health state’ defined by the EQ-5D-5L on a scale anchored at 0 (a health state considered as bad as death) and 1 (best health). Health states can be valued below 0, representing health states considered worse than death.

EQ-5D-5L data was collected at baseline (i.e. pre-surgery), in those who consented prospectively, and at 6 weeks, 6 months, and 12 months post-surgery in all participants. Index scores were generated using the mapping function developed by Hernández-Alava et al., utilising the *eq5dmap* command in Stata [19], as recommended by the National Institute for Health and Care Excellence [20]. Participants were also asked to complete a stoma questionnaire at 6 and 12 months to capture information about their stoma over time including whether they had their stoma closed. We categorised participants into PSH groups based on their responses to the question ‘Have you been told by a nurse or a doctor that you have a parastomal hernia?’ in the 12-month stoma questionnaire. The questionnaire allowed for 3 responses, ‘yes’, ‘no’, and ‘unknown’. We categorised all participants into 4 mutually exclusive groups based on responses to this question and other information provided in the 12-month questionnaire. The groups were: 1) reported no PSH; 2) reported having a PSH; 3) reported PSH status as unknown, left question blank, or did not complete any of the stoma questionnaire; and 4) reported their stoma closed.

### Analysis

All analyses were carried out in Stata 17 or 18. We used a repeated measures regression analysis (Stata command *xtmixed*) to capture the association between reporting PSH at 12 months and HRQoL measured by EQ-5D-5L at baseline, 6 weeks, 6 months, and 12 months. Reporting no PSH (group 1) was chosen as the reference group in the regression. We used the interaction of the time-point (baseline, 6 weeks, 6 months, and 12 months) and self-reported PSH incidence (group 2) to examine the magnitude of any decrement in HRQoL in those reporting PSH at 12 months. Our regression analysis included the following demographic, clinical, and other covariates: age at the time of surgery; gender; retrospective consent status; intended permanence of stoma; indication for surgery; stoma type; and surgery type. We compared the mean decrement in utility scores (No PSH—PSH) at 12 months to the standard deviation (0.161) of the normative utility scores from the UK population aged 60 to 64 (the mean age of the CIPHER cohort) [21]. Cohen’s D statistic was used to characterise any utility decrement associated with PSH as small (d ≥ 0.2 SDs), medium (d ≥ 0.5 SDs), or large (d ≥ 0.8 SDs) [22].

Using ordinal regression, we also calculated the odds ratios of reporting worse (higher-level) problems on each domain of the EQ-5D-5L in those who reported PSH (group 2 versus group 1) at 12 months post-surgery including the covariates listed above.

We calculated individual-level QALYs over the four time points based on the linear change assumption using the area under the curve method in the subset of the participants who had reported either no PSH or PSH (groups 1 and 2 only) [23]. Missing EQ-5D-5L index values were imputed using predictive mean matching (PMM) assuming that values were missing at random. Values were imputed using the *mi impute* command in a linear regression of EQ-5D-5L scores on PSH incidence during the year, controlling for the covariates listed above. Using a linear regression model, we estimated the quality adjusted life year (QALY) and quality adjusted life day (QALD) difference between those reporting PSH and not using the imputed dataset and the covariates listed above. As a sensitivity analysis, we also calculated QALYs and QALDs including only participants with complete EQ-5D-5L responses at all time points.

## Results

### Sample and baseline characteristics

**Fig. 1:**
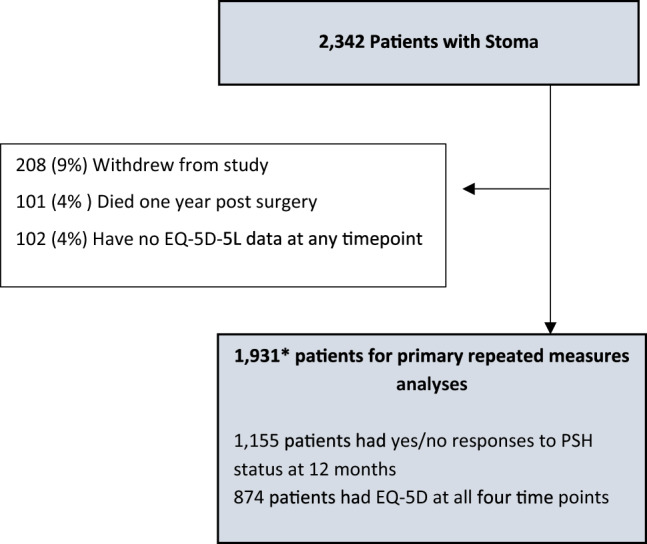
Patient Flow Diagram. *680 (35%) patients were retrospectively consented in the 6 weeks following surgery

At the time of analyses, CIPHER recruited 2,342 participants from 78 acute hospital trusts across the UK (Fig. 1). Of these, 101 (4%) died within 12 months, 208 (9%) withdrew consent for further data collection, and a further 102 (4%) did not complete EQ-5D-5L at any time point and were excluded from this analysis. A final dataset of 1,931 participants living with a stoma was analysed. At 12 months, 860 (44%) reported not having a PSH, 295 (15%) reported a PSH, 615 (32%) had unknown or missing PSH status, and 161 (8%) reported their stoma closed without reporting a PSH. The 1,931 participants completed the EQ-5D-5L on average 3.1 times out of a maximum of four time points; 874 completed the EQ-5D-5L at all four time points.

The mean age of participants was 63.5 years and 43% were female (Table 1). Consent was obtained retrospectively for 29% of participants. Colostomy surgery was performed in 58% of participants and ileostomy surgery in 42%. The majority (61%) had an end stoma and 36% had a loop stoma. The most common reason for stoma surgery was a malignant tumour (72%), followed by inflammatory bowel disease (IBD, 12%) with functional intestinal disorder and diverticular disease each comprising 5%.

**Table 1:** Baseline demographics and surgical characteristics by PSH Status

	PSH N = 295	No PSH N = 860	Unknown, Missing, or Stoma Closed N = 776	Total sample N = 1,931	p-value PSH vs. No PSH
Female	36%	47%	40%	43%	0.001^b^
Age, mean (SD)	66 (12)	63 (15)	62 (15)	63(15)	0.001^a^
Baseline EQ-5D-5L^d^, mean (SD)	0.773 (0.21)	0.766 (0.22)	0.742 (0.24)	0.755 (0.26)	0.695^a^
Indication for surgery					
Tumour- malignant	72%	70%	70%	70%	0.78^c^
IBD	7%	14%	13%	13%	
Diverticular disease	7%	4%	5%	5%	
Functional intestinal disorder	7%	5%	6%	6%	
Other/missing	6%	7%	6%	6%	
Surgery type (colostomy)	65%	63%	51%	58%	0.67^c^
Stoma type					
End	65%	72%	49%	62%	0.06^c^
Loop	32%	27%	48%	36%	
Other	3%	2%	3%	2%	
Permanent stoma	63%	66%	48%^e^	58%^e^	0.51^b^
Retrospective consent	29%	29%	29%	29%	0.98^b^

^a^T-test

^b^Chi2 test

^c^ANOVA (across all categories)

^d^Missing for those with retrospective consent

^e^10% missing

Individuals who reported a PSH were less likely to be female (36% vs. 47%; p < 0.01) and were older (mean age 66 vs. 63; p < 0.01) than those who reported no PSH (Table 1). There was a slightly greater percentage of loop stoma types in the PSH cohort (32% vs. 27%; p = 0.08). A slightly lower percentage of those in the PSH cohort had permanent stomas. However, there were no significant differences in the surgery type (colostomy or ileostomy), or in participants' baseline quality of life.

### PSH and health-related quality of life

**Fig. 2:**
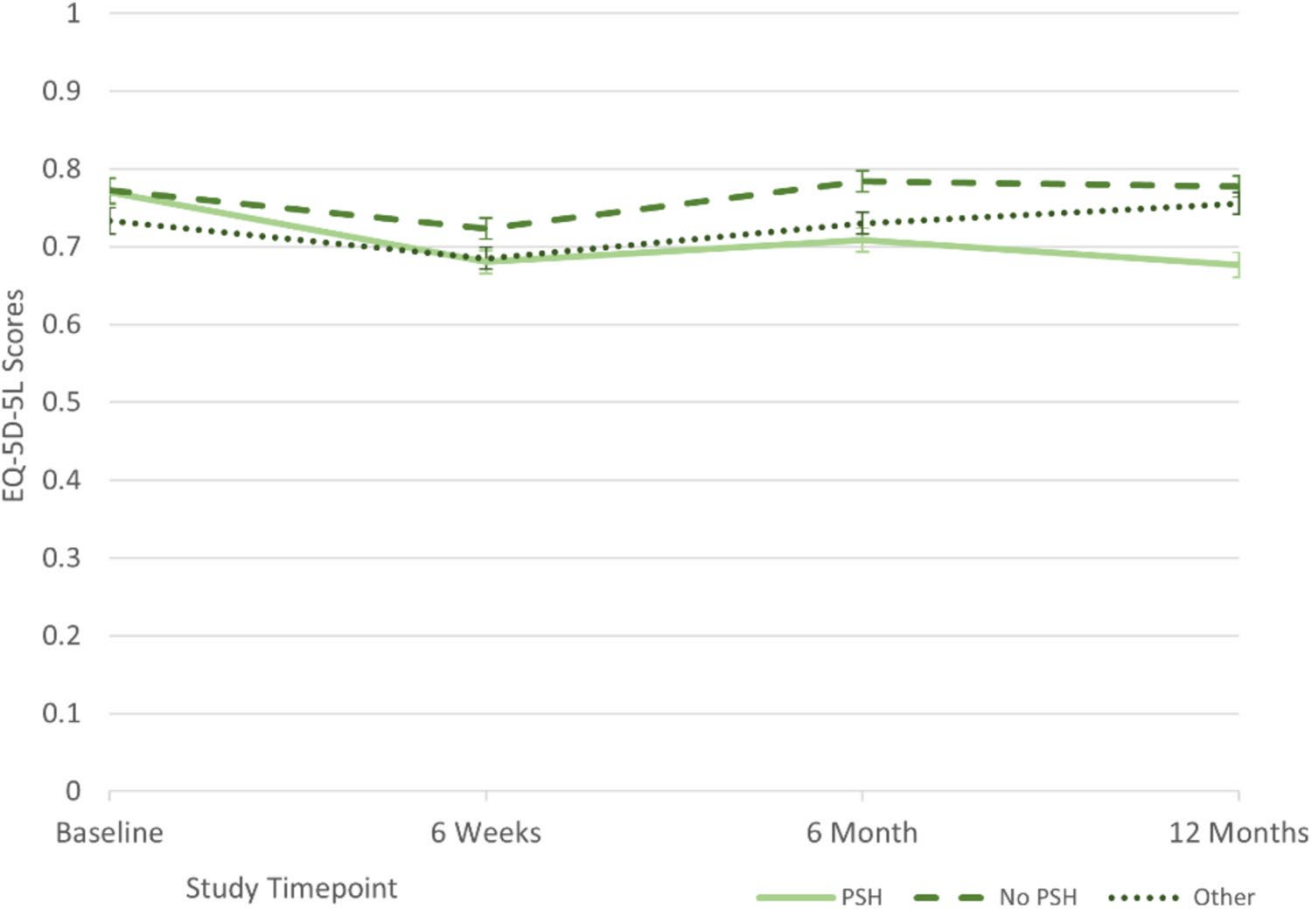
Mean EQ-5D-5L scores over time by PSH status at 12 months*. *N baseline = 1,427, 6 weeks 1,695, 6 months 1,615, 12 months 1,492; error bars represent 95% confidence intervals

Mean EQ-5D-5L scores in all three PSH status groups dropped between baseline and 6 weeks (Fig. 2) and then improved faster in the groups reporting no PSH or with unknown PSH status. The difference in HRQoL between those who reported PSH and no PSH at 12 months was evident at 6 weeks and continued to diverge up to 12 months.

When controlling for baseline patient characteristics and clinical variables in the repeated measures regression, we found the EQ-5D-5L scores were not significantly different at baseline between PSH and no PSH groups (−0.027 [CI: −0.057 to 0.003]). However, scores diverged between those reporting PSH and not, over the three follow-up periods (Table 2). Reporting a PSH at 12 months was associated with a worse EQ-5D-5L score of −0.043 [CI: −0.072 to −0.015] at 6 weeks, −0.075 [CI: −0.103 to −0.047] at 6 months and −0.099 [CI: −0.126 to −0.071] at 12 months. At 12 months, this difference represents a medium effect size based on calculation of the Cohen’s D statistic (d = 0.614).

**Table 2:** Association between HRQoL and self-reported PSH after adjusting for covariates N = 6,046 in 1,931 patients

	EQ5D-5L score increment/decrement	95% Confidence Interval
Timepoint (Ref: Baseline)		
6 weeks	−0.071**	−0.081 to −0.059
6 months	−0.019**	−0.030 to −0.007
12 months	−0.021**	−0.032 to −0.010
Interaction between timepoint and PSH reported at 12 M		
Baseline	−0.027	−0.057 to 0.003
6 weeks	−0.043**	−0.072 to −.015
6 months	−0.075**	−.103 to −.047
12 months	−0.099**	−.126 to −.071
Unk./missing PSH (Ref: No PSH)	−0.070**	−.090 to −.050
Stoma closed (Ref: No PSH)	0.026	−.007 to .059
Female	−0.017**	−.035 to −.001
Age	0.001**	.000 to .002
Indication (ref: Tumour)		
IBD	−0.042*	−.073 to −.010
Diverticular disease	−0.058**	−.098 to −.018
Functional intestinal disorder	−0.232**	−.270 to −0.193
Other	−0.193**	−0.227 to −0.158
Surgery type (Ref: Ileostomy)		
Colostomy	−0.044**	−0.065 to −0.023
Missing	0.034	−0.313 to 0.382
Stoma type (Ref: End)		
Loop	−0.037**	−0.064 to −0.010
Other	−0.070*	−0.128 to −0.012
Longevity (Ref: Permanent)		
Uncertain	0.029*	0.004 to 0.054
Missing	0.157**	0.045 to 0.270
Retrospective consent	0.011	−0.008 to 0.029
Constant	0.787**	0.735 to 0.839

Average of 3.1 completed EQ-5D-5L per participant

**Fig. 3:**
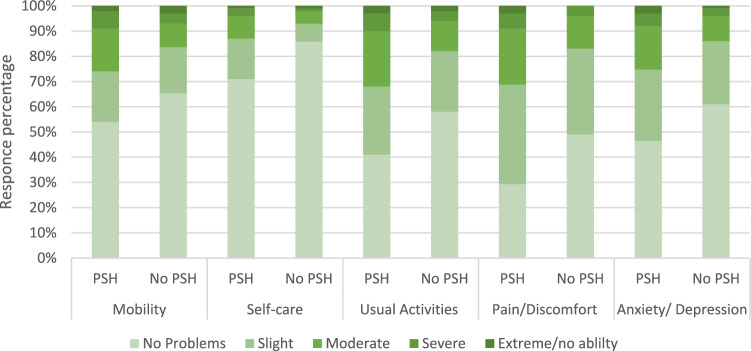
Self-reported problems by EQ-5D-5L domain and PSH status at 12 months (PSH n = 295, No PSH n = 860)

The greatest disparity in 12-month EQ-5D-5L responses was in the pain/discomfort domain (Fig. 3 and Table 3). Those reporting a PSH were more than twice as likely to report a higher level of pain/discomfort (odds ratio 2.80 [CI: 2.17 to 3.62]) at 12 months. They were also more than twice as likely to report greater difficulties with self-care (odds ratio 2.50 [CI: 1.80 to 3.47]), struggles with anxiety/depression (odds ratio 2.38 [CI: 1.82 to 3.10]), and greater challenges completing their usual activities (odds ratio 2.03 [CI: 1.57 to 3.91]). The least disparate domain was mobility (odds ratio 1.61 [CI: 1.23 to 2.10]).

**Table 3:** Odds ratios of reporting a higher (worse) response on each domain of the EQ-5D-5L at 12 months post-surgery among patients reporting PSH

EQ-5D-5L domain	Odds ratio (95% CI)
Mobility	1.61 (1.23–2.10)
Self-care	2.50 (1.80–3.47)
Usual activities	2.03 (1.57–2.61)
Pain/discomfort	2.80 (2.17–3.62)
Anxiety/depression	2.38 (1.82–3.10)

N = 1,126 patients with completed EQ-5D at 12 months and a Yes/No answer to PSH question at 12 months (n = 292 versus no PSH n = 834)

### PSH and QALYs

Of the 1,178 (61%) participants with “Yes or No” responses to the PSH question at 12 months, 496 (42%) individuals had missing EQ-5D-5L data at one or more time points. Imputation was used to estimate the 626 individual missing EQ-5D-5L scores and supplement the 4,085 complete responses. Linear regression on the imputed datasets demonstrated that those who reported PSH at 12 months, had 0.061 [CI: -0.082 to 0.040] fewer QALYs over the 12 months compared to those who did not (Supplementary Table 1). This equates to approximately 22.3 quality-adjusted life days (QALDs) lost each year. Sensitivity analysis using only complete case QALY data estimated a smaller QALY loss of −0.047 [CI: -0.072 to −0.024], amounting to 17.1 QALDs. (Supplementary Table 2).

## Discussion

The development of PSH is a common side effect in people living with a stoma. This analysis has shown a clear negative association between PSH and HRQoL. PSH was strongly associated with poorer scores across all dimensions of HRQoL captured by the EQ-5D-5L. Those reporting PSH were twice as likely to indicate greater pain/discomfort, anxiety/depression, difficulties with self-care, and usual activities. Mean quality of life across the whole sample showed an initial drop in quality of life between surgery and 6 weeks which improved at 6 months, then remained stable to 12 months. We found that the development of PSH was associated with 22.3 fewer QALDs in the first year post-surgery.

### Strengths and limitations

CIPHER is the largest prospective longitudinal study capturing HRQoL using the preference-based EQ-5D-5L. Data were collected from 78 hospital trusts across the UK and included a wide range of people living with stomas who had differing indications for surgery, surgery type, and varying methods of stoma formation. The longitudinal design of the study allowed us to identify changes in HRQoL over time. We were able to control for key clinical and demographic covariates that might otherwise confound the measured association between PSH and HRQoL. The observed association between PSH reported at 12 months and decreased HRQoL remained after adjustment for these covariates which strengthens the argument that the observed differences in HRQoL are causally related to PSH.

We categorised participants into PSH groups based on their self-reported PSH status at a single point in time (12 months). Self-reported PSH may differ from standardised clinical assessment of PSH or radiologically defined PSH, highlighting the differences between the surgeon-centred definitions of “true” parastomal hernia [6] and the more patient-centred definition of parastomal bulge that encompasses subdermal prolapse/siphon loop as well as true PSH [24]. However, the question specifically asked participants whether a health professional had informed them that they had developed a PSH; therefore, we believe that the results should reflect clinical judgments.

About one-third of participants did not self-report whether they had a PSH at 12 months and 8% of participants reported their PSH status as unknown. These participants with an uncertain PSH status also reported worse quality of life than those who reported no PSH. It is possible that our estimates of the decrement in HRQoL associated with PSH would have been different had the PSH status of these participants been known.

The EQ-5D-5L was completed on average at 3.1 out of the four time points. Some of the missing data were due to 29% of participants being retrospectively consented, so they could not complete the baseline measurement. However, not insignificant amounts of data were missing across all subsequent time points, including 84 participants who had missing EQ-5D-5L data across all time points. To mitigate this issue, we ran a repeated measures regression that makes use of all available EQ-5D-5L data at each time point. Furthermore, we used multiple imputation of EQ-5D-5L scores across all time points in our estimation of QALY decrements, taking advantage of all data available in our cohort.

The EQ-5D-5L was chosen as it is a generic quality of life measure that can be used to compare the relative effectiveness of treatments across health conditions. However, by design, the EQ-5D-5L scores will include health changes, for example co-morbidities, unrelated to the PSH, stoma or the underlying bowel problem. However, we would expect the prevalence of co-morbidities to be similar between groups (PSH vs no PSH) and therefore the relative difference observed will be predominantly related to the development of a PSH.

### Comparison with previous work

Previous trials investigating treatments to prevent or treat PSH have largely ignored HRQoL. Our work produced similar findings to other smaller non-randomised studies examining quality of life and PSH in different stoma populations. Van Dijk et al. [12] captured the quality of life using EQ-5D-3L and the short form 36 (SF-36) in a cross-sectional study of 139 end-ileostomy patients. Although an index score for EQ-5D-3L was not presented, a similar association was found between the development of PSH and the likelihood of reporting more severe pain/discomfort. However, unlike our work, no significant difference in the other four dimensions was found, potentially due to the smaller sample size. Differences across all four physical health dimensions (physical function, physical role, bodily pain, and general health) of the SF-36 were found between PSH groups; however, no significant differences were found in the mental health dimensions. Analysis of qualitative focus groups conducted by Krogsgaard et al., 2017 [25], explored the quality of life of those who experience a parastomal budge resulting from the formation of a permanent or temporary colostomy or ileostomy. The 20 participants highlighted discomforts including ‘unfamiliar bodily sensations’ and soreness and redness at the site of the stoma including painful stretching of the skin. Patients also highlighted that the ever-changing nature of the bulge was ‘transforming stoma care from a relatively simple to an increasingly difficult and complex task’. Patients in the Krogsgaard et al. [25] study also described the negative impact on body image, which the EQ-5D-5L does not directly assess, but may be reflected in the anxiety/depression domain. The ongoing PROPHER study will provide further evidence on quality of life after PSH repair [26]

### Implications for policy and future research

To our knowledge, this work provides the first estimates of the association between PSH and reduced utility scores and QALYs. The clear negative association of HRQoL and the incidence of PSH strengthens the case to develop surgical techniques, stoma care accessories, and treatments to reduce the incidence and severity of PSH as well as improve treatment when PSH occurs. Robust and generalisable estimates of HRQoL presented here can feed directly into modelling studies to assess the cost-effectiveness of such interventions within the stoma population [14, 15]. Treatments to reduce the impact of PSH may improve patients’ quality of life and reduce the burden of stoma treatment both to the health system and patients. Using the HRQoL estimates presented here, future research evaluating the cost-effectiveness of “watch and wait” vs. repair for PSH and the development of more effective surgical methods to repair PSH when it arises is possible. Repair of PSH can often be unsuccessful, and lead to multiple revision surgeries, lowering HRQoL and increasing the use of scarce resources, future research is needed to understand the resource use and cost implications of developing a PSH.

## Conclusion

This work demonstrates the significant impact PSH has across many dimensions of HRQoL. During the course of the year following surgery, those who developed PSH had 22.3 fewer quality-adjusted life days compared to those who avoided PSH. Consequently, therapies to prevent PSH have the potential to significantly improve patient health and reduce the cost associated with stoma care. The estimates of HRQoL presented here can be used in cost-effectiveness studies evaluating these therapies.

## Electronic supplementary material

Below is the link to the electronic supplementary material.

Supplementary file1 (DOCX 26 KB)

## Data Availability

It is our intention that data from the CIPHER study should be shared to benefit the kinds of patients who consented to participate. However, this manuscript has been written in advance of publishing manuscripts addressing the main objectives of the CIPHER study, i.e. to identify risk factors for PSH among patients with cancer, bowel disease, or other reasons for having a stoma. We are unable to share data from the study until these additional manuscripts have been accepted for publication, anticipated to be the end of June 2026. A full statement describing our position on data sharing is included in version 3.0 of our protocol, dated February 2024, available at: https://fundingawards.nihr.ac.uk/award/14/166/01

## Appendix

CIPHER core study team: Professor Jane Blazeby, Professor of Surgery, Bristol Medical School; Professor Chris Rogers, Professor of Medical Statistics and Clinical Trials, Bristol Trials Centre (BTC); Professor Thomas Pinkney, Professor of Surgery, University of Birmingham; Miss Natalie Blencowe, Consultant Senior Lecturer & MRC Clinician Scientist, Bristol Medical School; Professor Mark Callaway, Consultant Radiologist, University Hospitals Bristol NHS Foundation Trust; Mr Ian Daniels, Consultant Colorectal Surgeon, Royal Devon & Exeter NHS Foundation Trust; Mrs Amanda Gunning, Lead Stoma Care Nurse, Royal Devon & Exeter NHS Foundation Trust; Mr Angus McNair, Consultant Senior Lecturer & NIHR Clinician Scientist, Bristol Medical School; Ms Hana Tabusa, Clinical Trials Coordinator, Bristol Trials Centre (BTC); Miss Charlotte Murkin, Research Fellow, Bristol Medical School. **CIPHER study investigators: Research team members who contributed to obtaining funding for the study or to managing its conduct**: BLAZEBY, Jane M; PINKNEY, Thomas; ROGERS, Chris A; TABUSA, Hana; McNAIR, Angus; CALLAWAY, Mark; BLENCOWE, Natalie B. Aintree University Hospitals NHS Foundation Trust: ARTHUR, James (PI). Ashford and St Peter's Hospitals NHS Foundation Trust: BEARN, Philip (PI); KUMAR, Lalit; MADANI, Rana; NISAR, Pasha Javed; THOMAS, Gregory Paul. Barking, Havering and Redbridge University Hospitals NHS Trust: HUANG, Joe (PI). Basildon and Thurrock University Hospitals NHS Foundation Trust: LOVETT, Bryony (PI); ASHRAF, Nadeem; KHAN, Laeeq Ur Rahman. Blackpool Teaching Hospitals NHS Foundation Trust: BLACKMORE, Alexander (PI). Calderdale and Huddersfield NHS Foundation Trust: COWLEY, Jonathan (PI); GREY, Tamsyn Jane Olivia; JUNEJO, Muneer Ahmed. Cambridge University Hospitals NHS Foundation Trust: FERNHEAD, Nicola (PI); WHEELER, James Malcolm Donald; POWAR, Michael Paramjit; HALL, Nigel Ruthven; PANAGIOTOPOULOU, Ioanna Georgiou. Chelsea and Westminster Hospital NHS Foundation Trust: WARREN, Oliver (PI); MILLS, Sarah Catherine. Chesterfield Royal Hospital NHS Foundation Trust: NARULA, Harjeet (PI); SARGEN, Kevin Christie NHS Foundation Trust: PARMAR, Kat (PI); SUTTON, Paul (associate PI). City Hospitals Sunderland NHS Foundation Trust: ROYLE, James (PI); HOLTHAM, Stephen John; O'DAIR, Graham Nelson. Colchester Hospital University NHS Foundation Trust: ARULAMPALAM, Tan (PI). Countess of Chester Hospital NHS Foundation Trust: VIMALACHANDRAN, Dale (PI); EARDLEY, Nicola Jayne. Croydon Health Services NHS Trust: MOODY, Debbie (PI); MOHAMED, Said Abdirahman. Derby Teaching Hospital NHS Foundation Trust: BHALLA, Ashish (PI). Doncaster & Bassetlaw Teaching Hospitals NHS Foundation Trust: WILSON, Tim (PI). Dorset County Hospital NHS Foundation Trust: MARCH, Catherine (PI); NG, Paul Wing Kay; TALWAR, Anjay. East Cheshire NHS Trust: SADAT, Mohammed (PI); KHAN, Usman Ameer; SMART, Christopher Jeremy. East Kent Hospitals University NHS Foundation Trust: EVANS, Jessica (PI); KHUSHAL, Amjad; BUGREN, Seeraj Mustafa; BENZIGER, Harrison; MANGAM, Sudhakar. East Lancashire Hospitals NHS Trust: JONES, Lyndon (PI); GIL, Michael David. East and North Hertfordshire NHS Trust: LLOYD, Geraint (PI); REAY-JONES, Nicholas Havelock John; ATKIN, Gary Keith; GUPTA, Vivek; TEWARI, Shirish. Gateshead Health NHS Foundation Trust: O’LOUGHLIN, Paul (PI). Glasgow Royal Infirmary (NHS Greater Glasgow and Clyde): PARK, James (PI). Gloucestershire Hospitals NHS Foundation Trust: MURRAY, Catherine (PI). Great Western Hospitals NHS Foundation Trust: PULLEN, Judy (PI); OWAIS, Anwar E. A.; ALEXANDER, Roderick James; LIM, Jeffrey Wei Wen; THORN, Christopher Charles. Heart of England NHS Foundation Trust: YOUSSEF, Haney (PI). Imperial College Healthcare NHS Trust: KINROSS, James (PI); REESE, George; MURPHY, Jamie. Ipswich Hospital NHS Trust: PITT, James (PI); EL-BEIALY, Hesham Abd-El-Moneim Abd-El-Meguid; MALIK, Ali Irqam; MALIK, Arshad Hussain; CRABTREE, Michael David. Kingston Hospital NHS Foundation Trust: TUDYKA, Vera (PI); BLOOM, Ian Tobias Michael. Leeds Teaching Hospitals NHS Trust: TIERNAN, Jim (PI); RIYAD, Kallingal; JAYNE, David George; DA SILVA, Nigel Fortunato Xavier. London North West Healthcare NHS Trust: WARUSAVITARNE, Janindra (PI). Medway NHS Foundation Trust: GRIMES, Caris (PI). Mid Cheshire Hospitals NHS Foundation Trust: GABRIEL, Claire (PI); BRUCE, Caroline Anne; HARDMAN, Jonathan Gordon; NOCKOLDS, Claire Louise. Manchester University NHS Foundation Trust (Manchester Royal Infirmary): HORNUNG, Ben (PI); STYLIANIDES, Nicholas Andreas. Manchester University NHS Foundation Trust (Wythenshawe Hospital): DUFF, Sarah (PI); TELFORD, Karen Jane; BARAZA, Wasilwa. Mid Yorkshire Hospital NHS Trust: WELBOURN, Hannah (PI); FAWOLE, Adeshina Sergei (associate PI); MOHAMED BASHEER, Abdul Majeed; SIVAKUMAR, Rangasamy; THOMSON, Helen Jane; NOFAL, Emma Mary. Morriston Hospital, Swansea (ABM University Health Board): TAYLOR, Greg (PI); BEDFORD, Matthew Robert; HARRIES, Rhiannon Louise; HARRIS, Dean Anthony; EVANS, Martyn Dominic. Newcastle Upon Tyne Hospitals NHS Foundation Trust: BRADY, Richard (PI); COYNE, Peter Edward; GALLAGHER, Hugh Joseph. Norfolk and Norwich University Hospitals NHS Foundation Trust: HERNON, James (PI); SHAIKH, Irshad Ahammed Abdul Khadar; PAL, Atanu; STEARNS, Adam Timothy; KAPUR, Sandeep. North Bristol NHS Trust: LYONS, Ann (PI); SUMRIEN, Haytham Ali; PULLYBLANK, Anne Marie; BURT, Caroline Grace. North Tees and Hartlepool NHS Foundation Trust: ETHERSON, Kevin (PI); GILL, Talvinder Singh. North West Anglia NHS Foundation Trust: MAKHIJA, Rohit (PI); DENNIS, Robert James. Nottingham University Hospitals NHS Trust: ACHESON, Austin (PI); BHARATHAN, Balamurali. Oxford University Hospitals NHS Foundation Trust: PAINTON, Jodie (PI); LANE, Jessica (associate PI); LYDIAT, Sarah; MOTTRAM, Katherine Plymouth Hospitals NHS Trust: COLEMAN, Mark (PI); SMOLAREK, Sebastian Krzysztof; BRUNDELL, Simeon Mark. Poole Hospital NHS Foundation Trust (as of October 2020 University Hospitals Dorset NHS Foundation Trust): NASH, Guy (PI); AHMAD, Mukhtar Ibrahim; QURESHI, Tahseen Iftikhar; MUREB, Amro Ahmed Abdullatif. Portsmouth Hospitals NHS Trust: KHAN, Jim (PI); CONTI, John Antony. Queen Elizabeth Hospital King's Lynn NHS Foundation Trust: HARDING, Melanie (PI); HYDER, Syed Answer; EASTERBROOK, Jonathan Richard; CULLEN, Paul Thomas; KHAN, Zulfiqar Ali Jehangir. Raigmore Hospital, Inverness (NHS Highland): OLIPHANT, Raymond (PI); RICHARDS, Colin Hewitt. Royal Alexandra Hospital, Paisley (NHS Greater Glasgow & Clyde): MOUG, Susan (PI); CRAWFORD, James (associate PI); RENWICK, Andrew Austin. Royal Bolton Hospital NHS Foundation Trust: FAULKNER, Gemma (PI); HARRIS, Richard Paul; POLLARD, James Scott. Royal Cornwall Hospitals NHS Trust: MAY, Denzil (PI); FELDMAN, Melanie Ann. Royal Devon and Exeter NHS Foundation Trust: SMART, Neil (PI and author); GEE, Andrew Stephen; DANIELS, Ian Richard; BETHUNE, Robert Mackenzie; MANSFIELD, Stephen David Royal Surrey County Hospital NHS Foundation Trust: SCALA, Andrea (PI). Royal Wolverhampton NHS Trust: YASSIN, Nuha A (PI); BADGER, Ian Laurence Russell; ELGADDAL, Sanaa Abdalla Hassan.Salisbury NHS Foundation Trust: BRANAGAN, Graham (PI); WEST, Charles (associate PI); PADWICK, Robert Thomas; SZYMANKIEWICZ, Mark Andrew; SAMAR, Ali Murtaza. Sherwood Forest Hospitals NHS Foundation Trust: BADRINATH, Krishnamurthy (PI); WATSON, Nicholas Francis Scot. Stockport NHS Foundation Trust: HALL, Claire (PI); HUMAYUN, Qasim Mohammad. St Helens and Knowsley Teaching Hospitals NHS Trust (Whiston Hospital): SAMAD, Ajai (PI); JAVED, Muhammad Ahsan (associate PI). Taunton and Somerset NHS Foundation Trust: MACKEY, Paul (PI); EDWARDS, Thomas James. University Hospitals Birmingham NHS Foundation Trust: RICHARDSON, Jonathan (PI); MALIK, Kamran Ishaque; ADDISON, Sarah Louise; ASHRAF, Shazad; OLIVEIRA CUNHA, Melissa. University Hospitals Derby and Burton NHS FT (Queen’s Hospital): VAKIS, Stelios (PI); THOMAS, Pradeep Franklin. Salford Royal NHS Foundation Trust: SLADE, Dominic (PI); PEARCE, Lyndsay Ellen Sheffield Teaching Hospitals NHS Foundation Trust: BROWN, Steven (PI); SKINNER, Paul Patrick; DAVIS, George Roderick Shorland; AMIN, Shwan Niazi Omar; LOGANATHAN, Arunan. Tameside and Glossop Integrated Care NHS Foundation Trust: MUHAMMAD, Karim (PI). United Lincolnshire Hospitals NHS Trust: RAO, Milind (PI). University Hospitals Coventry and Warwickshire NHS Trust: LEONG, Kai (PI); BAJWA, Adeel Ahmad; EVANS, Charles Francis Mitchell; WONG, Ling Sen; QURESHI, Adnan Ali. University Hospitals Bristol and Weston NHS Foundation Trust (formally University Hospitals Bristol NHS Foundation Trust): SHABBIR, Jamshed (PI); MESSENGER, David Edward; LONGMAN, Robert John; RANDALL, Jonathan Keith; TAYLOR, Mark. University Hospitals of Leicester NHS Trust: CHAUDHRI, Sanjay (PI); SINGH, Baljit. University Hospitals Southampton NHS Foundation Trust: MIRNEZAMI, Alex (PI); NICHOLS, Paul Henry. University Hospital of Wales, Cardiff: ANSELL, James (co-PI); DAVIS, Leigh (co-PI), CORNISH, Julie Ann; HORWOOD, James Peter; DAVIES, Michael Morgan; TORKINGTON, Jared. University Hospitals of Morecambe Bay NHS Foundation Trust: RAYMOND, Thomas (PI); DAVIES, Emma Jane; PATEL, Panna Kantibhai; RYSKA, Ondrej. Warrington and Halton Hospitals NHS Foundation Trust: DITCHFIELD, Lisa (PI); GOSSEDGE, Gemma (previous PI). Western General Hospital, Edinburgh (NHS Lothian): SPEAKE, Doug (PI); COLLIE, Mhairi Helen Stewart. Wirral University Teaching Hospital NHS Foundation Trust: WALSH, Ciaran (PI); SMITH, Darren Jeffrey. Worcestershire Acute Hospitals NHS Trust: NICOL, Deborah (PI); PANDEY, Steve Bahadur. Wrightington, Wigan and Leigh NHS Foundation Trust: MACKENZIE, Naomi (PI). Yeovil District Hospital NHS Foundation Trust: DALTON, Richard (PI); FRANCIS, Nader Kamal; ALLISON, Andrew Scott.

## References

[1] NHS.uk: Colostomy. https://www.nhs.uk/conditions/colostomy/ Accessed 25/03/2021 2021.

[2] Colostomy UK: What is a stoma? . https://www.colostomyuk.org/information/what-is-a-stoma/ Accessed 25/03/21 2021.

[3] Snowsill, T., Huxley, N., Hoyle, M., Jones-Hughes, T., Coelho, H., Cooper, C., et al.: A systematic review and economic evaluation of diagnostic strategies for Lynch syndrome. Health Technol. Assess. **18**(58), 1–406 (2014). 10.3310/hta18580 25244061 10.3310/hta18580 PMC4781313

[4] Stoma Care Nurses High Impact Action Steering Group High Impact Actions for Stoma Care. In: Coloplast, editor. Apollo Nurisng2010.

[5] Black, P.: Stoma care nursing management: cost implications in community care. Br. J. Community Nurs. **14**(8), 350 (2009). 10.12968/bjcn.2009.14.8.43515 19684556 10.12968/bjcn.2009.14.8.43515

[6] Smietanski, M., Szczepkowski, M., Alexandre, J.A., Berger, D., Bury, K., Conze, J., et al.: European Hernia Society classification of parastomal hernias. Hernia **18**(1), 1–6 (2014). 10.1007/s10029-013-1162-z 24081460 10.1007/s10029-013-1162-z PMC3902080

[7] Lopez-Cano, M., Lozoya-Trujillo, R., Quiroga, S., Sanchez, J.L., Vallribera, F., Marti, M., et al.: Use of a prosthetic mesh to prevent parastomal hernia during laparoscopic abdominoperineal resection: a randomized controlled trial. Hernia **16**(6), 661–667 (2012). 10.1007/s10029-012-0952-z 22782367 10.1007/s10029-012-0952-z

[8] Pilgrim, C.H., McIntyre, R., Bailey, M.: Prospective audit of parastomal hernia: prevalence and associated comorbidities. Dis. Colon Rectum **53**(1), 71–76 (2010). 10.1007/DCR.0b013e3181bdee8c 20010354 10.1007/DCR.0b013e3181bdee8c

[9] Hotouras, A., Murphy, J., Thaha, M., Chan, C.L.: The persistent challenge of parastomal herniation: a review of the literature and future developments. Colorectal Dis. **15**(5), e202–e214 (2013). 10.1111/codi.12156 23374759 10.1111/codi.12156

[10] Londono-Schimmer, E.E., Leong, A.P., Phillips, R.K.: Life table analysis of stomal complications following colostomy. Dis. Colon Rectum **37**(9), 916–920 (1994). 10.1007/BF02052598 8076492 10.1007/BF02052598

[11] Ripoche, J., Basurko, C., Fabbro-Perray, P., Prudhomme, M.: Parastomal hernia. A study of the French federation of ostomy patients. J. Visc. Surg. **148**(6), 435–441 (2011). 10.1016/j.jviscsurg.2011.10.006 10.1016/j.jviscsurg.2011.10.006 22130074

[12] van Dijk, S.M., Timmermans, L., Deerenberg, E.B., Lamme, B., Kleinrensink, G.J., Jeekel, J., et al.: Parastomal Hernia: Impact on Quality of Life? World J. Surg. **39**(10), 2595–2601 (2015). 10.1007/s00268-015-3107-4 26216640 10.1007/s00268-015-3107-4

[13] Mohiuddin, S., Hollingworth, W., Rajaretnam, N., Reeves, B.C., Smart, N.J.: Use of prophylactic mesh during initial stoma creation to prevent parastomal herniation: a systematic review and meta-analysis of randomised controlled trials. Colorectal Dis. **23**(11), 2821–2833 (2021). 10.1111/codi.15849 34331836 10.1111/codi.15849

[14] Lee, L., Saleem, A., Landry, T., Latimer, E., Chaudhury, P., Feldman, L.S.: Cost effectiveness of mesh prophylaxis to prevent parastomal hernia in patients undergoing permanent colostomy for rectal cancer. J. Am. Coll. Surg. **218**(1), 82–91 (2014). 10.1016/j.jamcollsurg.2013.09.015 24210147 10.1016/j.jamcollsurg.2013.09.015

[15] Mohiuddin, S., Reeves, B.C., Smart, N.J., Hollingworth, W.: A semi-Markov model comparing the lifetime cost-effectiveness of mesh prophylaxis to prevent parastomal hernia in patients undergoing end colostomy creation for rectal cancer. Colorectal Dis. **23**(11), 2967–2979 (2021). 10.1111/codi.15848 34331840 10.1111/codi.15848

[16] Tabusa, H., Blazeby, J.M., Blencowe, N., Callaway, M., Daniels, I.R., Gunning, A., et al.: Protocol for UK Cohort Study to Investigate the Prevention of Parastomal Hernia: (The CIPHER Study). Colorectal Dis. (2021). 10.1111/codi.15621 33686656 10.1111/codi.15621

[17] NCEPOD The NCEPOD Classification of Interventions. https://www.ncepod.org.uk/classification.html# Accessed 25/03/21 2021.

[18] Herdman, M., Gudex, C., Lloyd, A., Janssen, M., Kind, P., Parkin, D., et al.: Development and preliminary testing of the new five-level version of EQ-5D (EQ-5D-5L). Qual. Life Res. **20**(10), 1727–1736 (2011). 10.1007/s11136-011-9903-x 21479777 10.1007/s11136-011-9903-x PMC3220807

[19] Monica Hernandez, A., Pudney, S.: A command for mapping between EQ-5D-3L and EQ-5D-5L. Stata J. **18**, 395–415 (2018)

[20] van Hout, B., Janssen, M.F., Feng, Y.S., Kohlmann, T., Busschbach, J., Golicki, D., et al.: Interim scoring for the EQ-5D-5L: mapping the EQ-5D-5L to EQ-5D-3L value sets. Value Health. **15**(5), 708–715 (2012). 10.1016/j.jval.2012.02.008 22867780 10.1016/j.jval.2012.02.008

[21] McNamara, S., Schneider, P.P., Love-Koh, J., Doran, T., Gutacker, N.: Quality-adjusted life expectancy norms for the English population. Value Health **26**(2), 163–169 (2023). 10.1016/j.jval.2022.07.005 35965226 10.1016/j.jval.2022.07.005

[22] Lakens, D.: Calculating and reporting effect sizes to facilitate cumulative science: a practical primer for t-tests and ANOVAs. Front. Psychol. **4**, 863 (2013). 10.3389/fpsyg.2013.00863 24324449 10.3389/fpsyg.2013.00863 PMC3840331

[23] Manca, A., Hawkins, N., Sculpher, M.J.: Estimating mean QALYs in trial-based cost-effectiveness analysis: the importance of controlling for baseline utility. Health Econ. **14**(5), 487–496 (2005). 10.1002/hec.944 15497198 10.1002/hec.944

[24] Krogsgaard, M., Gogenur, I., Helgstrand, F., Andersen, R.M., Danielsen, A.K., Vinther, A., et al.: Surgical repair of parastomal bulging: a retrospective register-based study on prospectively collected data. Colorectal Dis. **22**(11), 1704–1713 (2020). 10.1111/codi.15197 32548884 10.1111/codi.15197

[25] Krogsgaard, M., Thomsen, T., Vinther, A., Gogenur, I., Kaldan, G., Danielsen, A.K.: Living with a parastomal bulge - patients’ experiences of symptoms. J. Clin. Nurs. **26**(23–24), 5072–5081 (2017). 10.1111/jocn.14009 28793391 10.1111/jocn.14009

[26] Group PSC: The PROPHER study: patient-reported outcomes after parastomal hernia treatment-a prospective international cohort study. Colorectal Dis. **26**(3), 554–563 (2024). 10.1111/codi.16859 38296915 10.1111/codi.16859

