# Supplementary material for: The impact of parastomal hernia on quality of life using data from the CIPHER prospective cohort study
Source: Eur J Health Econ. 2025 Mar 11;26(7):1209–18. doi: 10.1007/s10198-025-01768-5 (PMC12432048; doi:10.1007/s10198-025-01768-5)
Supplement: Supplementary file 1 — Supplementary file1 (DOCX 26 KB) [file 10198_2025_1768_MOESM1_ESM.docx]

Supplementary Material

# The impact of parastomal hernia on quality of life using data from the CIPHER prospective cohort study.

Joel Glynn^1*^, William Hollingworth^1^, Jessica Harris^2^, Syed Mohiuddin^3^, Lucy Ellis ^2^,Barnaby C Reeves^2^, Neil Smart^4^ on behalf of the CIPHER study group^4^

Supplementary Table 1 QALY analyses Imputed Dataset

| Variable | Coefficient | 95% Confidence Interval |
| --- | --- | --- |
|  |  |  |
| PSH (yes) | -0.06 | -0.08 to -0.04 |
| Age | 0.00 | 0.00 to 0.02 |
| Sex (female) | 0.01 | -0.01 to 0.00 |
| Stoma type (reference: End) |  |  |
| Loop | -0.12 | -0.01 to 0.03 |
| Other | -0.02 | -0.91 to 0.05 |
| Indication for Surgery  (reference: Tumour Malignant) |  |  |
| Inflammatory Bowel Disease | 0.44 | 0.01 to 0.08 |
| Diverticular Disease | 0.58 | 0.07 to 0.11 |
| Functional Intestinal Disorder | -0.05 | -0.10 to -0.01 |
| Other | -0.08 | -0.12 to -0.04 |
| Missing | -0.18 | -0.61 to 0.25 |
| Longevity (Reference: Permanent) |  |  |
| Uncertain | 0.03 | -0.00 to 0.06 |
| Missing | -0.17 | -0.61 to 0.25 |
| Surgery Type (Reference: Ileostomy) |  |  |
| Colostomy | 0.00 | -0.02 to 0.23 |
| Baseline EQ-5D-5L utility score | 0.58 | 0.53 to 0.63 |
| Constant | 0.24 | 0.17 to 0.31 |

Supplementary Table 2 QALY analyses complete case

| Variable | Coefficient | 95% Confidence Interval |
| --- | --- | --- |
|  |  |  |
| PSH (yes) | -0.05 | -0.07 to -0.02 |
| Age | 0.00 | 0.00 to 0.00 |
| Sex (female) | 0.02 | -0.00 to 0.04 |
| Stoma type (reference: End) |  |  |
| Loop | -0.01 | -0.05 to 0.02 |
| Other | -0.02 | -0.1 to 0.06 |
| Indication for Surgery  (reference: Tumour Malignant) |  |  |
| Inflammatory Bowel Disease | 0.07 | 0.03 to 0.11 |
| Diverticular Disease | 0.07 | 0.01 to 0.13 |
| Functional Intestinal Disorder | -0.04 | -0.09 to 0.01 |
| Other | -0.10 | -0.14 to -0.05 |
| Missing | -0.02 | -0.29 to 0.25 |
| Longevity (Reference: Permanent) |  |  |
| Uncertain | 0.03 | 0.00 to 0.06 |
| Missing | - | - |
| Surgery Type (Reference: Ileostomy) |  |  |
| Colostomy | 0.01 | -0.02 to 0.04 |
| Baseline EQ-5D-5L utility score | 0.64 | 0.58 to 0.70 |
| Constant | 0.19 | 0.11 to 0.27 |
